# Neural mechanisms of adaptive behavior: Dissociating local cortical modulations and interregional communication patterns

**DOI:** 10.1016/j.isci.2024.110995

**Published:** 2024-09-20

**Authors:** Nasibeh Talebi, Astrid Prochnow, Christian Frings, Alexander Münchau, Moritz Mückschel, Christian Beste

**Affiliations:** 1 Cognitive Neurophysiology, Department of Child and Adolescent Psychiatry, Faculty of Medicine, TU Dresden, 01309 Dresden, Germany; 2 Cognitive Psychology, University of Trier, 54269 Trier, Germany; 3 Institute of Systems Motor Science, University of Lübeck, 23562 Lübeck, Germany

**Keywords:** Behavioral neuroscience, Cognitive neuroscience

## Abstract

Adaptive behavior is based on flexibly managing and integrating perceptual and motor processes, and the reconfiguration thereof. Such adaptive behavior is also relevant during inhibitory control. Although research has demonstrated local activity modulations in theta and alpha frequency bands during behavioral adaptation, the communication of brain regions is insufficiently studied. Examining directed connectivity between brain regions using a machine learning approach, a generally increased activity, but decreased connectivity within a temporo-occipital theta band network was revealed during the reconfiguration of perception-action associations during inhibitory control. Additionally, a fronto-occipital alpha-theta interplay yielded a decrease in directed connectivity during reconfiguration processes, which was associated with lower error rates in behavior. Thus, adaptive behavior relies on both local increases and decreases of activity depending on the frequency band, and concomitant decreases in communication between frontal and sensory cortices. The findings reframe common conceptualizations about how adaptive behavior is supported by neural processes.

## Highlights

• Increased event file overlap linked to theta and lower alpha/beta band activity
• Network connectivity strength weakens as event file overlap increases
• Adaptive behavior tied to local activity changes and reduced directed connectivity

## Graphical abstract

**Figure:**
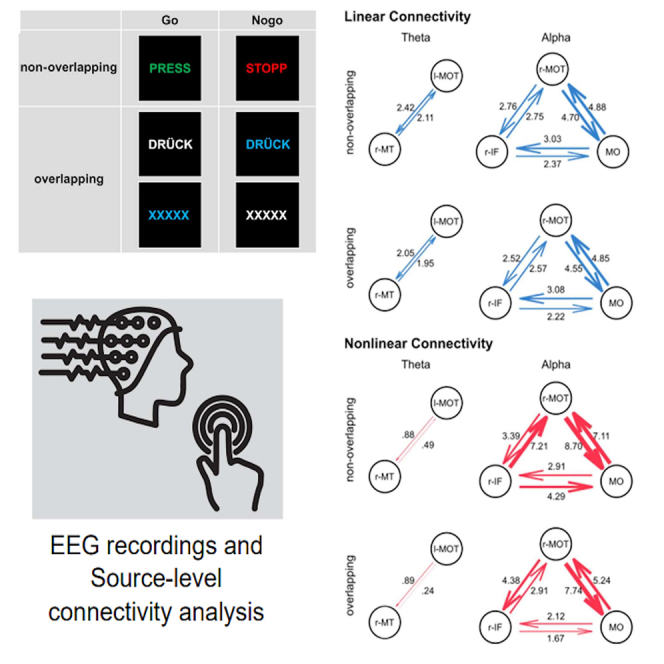

## Introduction

The integration of perception and action into seemingly effortless goal-directed behavior is a main asset of the central nervous system. These processes have been framed by the Theory of Event Coding (TEC)^1^ and its more recent derivatives,^2^^,^^3^ which consider neurophysiological processes.^4^ These theories assume that close associations (bindings) exist between features defining the stimulus and features defining the motor processes once the response must be executed after perceiving the stimulus. These bindings are stored in so-called event files.^5^ Problems arise whenever the identical action has to be executed (or inhibited) based on an altered sensory input, or when nearly identical sensory input triggers different actions.^6^ Under these circumstances, performance (i.e., response speed and/or accuracy) declines because the event file must be reconfigured. In the context of response inhibition, the false alarm rate has been shown to be increased when features (e.g., color) are shared between stimuli indicating to execute a response and stimuli indicating to inhibit the response.^7^^,^^8^^,^^9^ These findings imply that additional behavioral adaptions may be necessary during the course of response inhibition.^7^^,^^8^^,^^9^ The neurophysiological processes underlying this are increasingly better understood,^10^^,^^11^ and it seems that different aspects of perception-action integration are reflected in distinct frequency bands: the theta frequency band appears to be relevant to bind/build and retrieve event files, the alpha frequency band likely reflects top-down and bottom-up processes influencing these processes, and the beta frequency band is thought to be associated with the maintenance of an established event file structure.^4^ Event files are processed in a distributed fashion^7^^,^^12^^,^^13^ with occipito-parietal and frontal cortical regions being particularly important.^14^^,^^15^^,^^16^^,^^17^^,^^18^ Importantly, network-like, distributed processes suggest that there is a directed transfer of information between brain regions during perception-action integration. The relevance of considering such an information transfer between brain regions is underlined by the recent spatiotemporal neuroscience approach,^19^^,^^20^ as it requires the concomitant consideration of the temporal relationship of brain signals as well. Whether such an information transfer is evident in how the information transfer is organized during perception-action integration is elusive.

It needs to be considered that directed functional connectivity (which will be called short “directed connectivity”) between brain regions is probably not only linear, but also nonlinear.^21^^,^^22^^,^^23^^,^^24^ Moreover, the integration of perception and action depends on feedforward and feedback loops,^25^^,^^26^ in which linear and non-linear dynamic plays a role.^27^^,^^28^^,^^29^ Therefore, we examine the relative contribution of linear and nonlinear organizations of directed connectivity between cortical regions during perception-action integration in response inhibition, considering the theta, alpha, and beta frequency bands. To this end, we employ a machine learning approach (nonlinear Causal Relationship Estimation by Artificial Neural Network, nCREANN^30^^,^^31^). Considering TEC, a counter-intuitive prediction is that directed connectivity between brain regions should decrease when it is necessary to reconfigure an event file. This is because event file processing reflects dynamics in a network and event file reconfiguration is inefficient when bindings between stimulus and response features are strong.^6^^,^^32^ However, these processes need to be controlled.^4^ Therefore, connections in a network must be weakened whenever reconfigurations of event files are necessary to allow the integration of new information. With respect to the control of these processes (e.g., via alpha band activity),^4^ it is possible that especially networks supporting alpha-band related control are increased in their directed connectivity. This directed network hypothesis contrasts with common notions according to which increases in activity (e.g., in the theta band) are observed when there is a need to engage in cognitive control with the goal of adapting behavior.^33^ However, it is still possible that there are increases in activity that do not affect the strength of directed connectivity between cortical regions in a network. If this is the case this would suggest that adaptive behavior relies on concomitantly occurring opposing processes: (i) local increases of activity in certain cortical regions, and (ii) a concomitant decrease in directed connectivity between these regions.

## Results

### Behavioral performance

**Figure 1:**
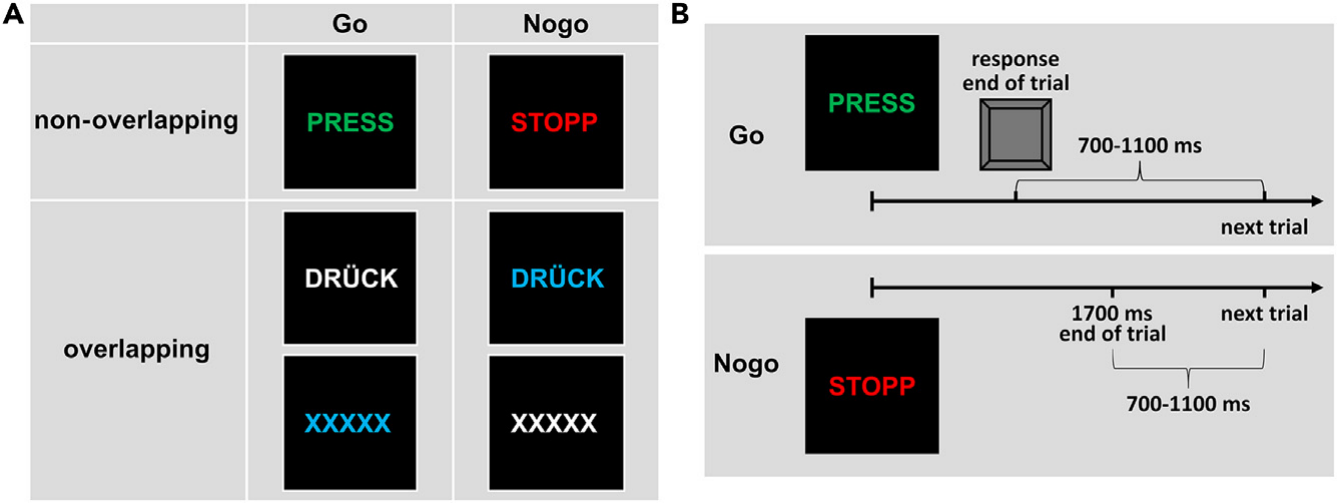
Task Figure part (A) shows the stimuli used in the task separately for Go and Nogo conditions and non-overlapping and overlapping conditions, respectively. Figure part (B) depicts the course and timing of a correct trial (top: Go trial; bottom: Nogo trial) on the example of the stimuli of the non-overlapping condition.

**Figure 2:**
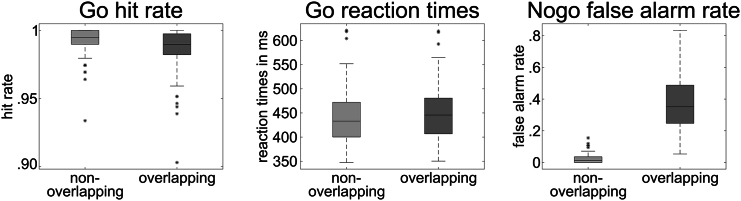
Distribution of the behavioral data Boxplot graphs for hit rates in Go trials, reaction times in Go trials, and false alarm rates in Nogo trials (from left to right) separated for non-overlapping and overlapping conditions. The non-overlapping condition is shown in lighter coloring than the overlapping condition. The horizontal line within the box depicts the median. Outliers (more than two standard deviations above or below the median) are denoted as an asterisk.

The participants conducted a modified Go/Nogo task the stimuli of which were created according to TEC, resulting in one condition in which features between Go and Nogo stimuli overlapped (overlapping condition) and another condition in which Go and Nogo stimuli were completely distinct (non-overlapping condition) (see Figure 1 for more information). Thus, the task measured inhibition performance as well as the (re)binding of event files. The distribution of the behavioral data is displayed in Figure 2. Concerning the Go hit rate, a paired t-test revealed significantly decreased hit rates in the overlapping (98.5 ± 1.7%) compared to the non-overlapping (99.2 ± 1.1%) Go condition (t(78) = 5.39, *p* < 0.001, d = 0.607). For the Go RTs, RTs were significantly increased in the overlapping (452 ± 57 ms) compared to the non-overlapping Go condition (441 ± 56 ms; t(78) = −4.48, *p* < 0.001, d = −0.504). Importantly, the Nogo false alarm rate was significantly increased in the overlapping (37.3 ± 17.1%), compared to the non-overlapping Nogo condition (2.3 ± 3.3%; t(78) = −20.51, *p* < 0.001, d = −2.308). As none of the behavioral differences was affected by gender (main effect of *Gender*: F(1,77) ≤ 2.00, *p* ≥ 0.161; interaction of *Overlap* and *Gender*: F(1,77) ≤ 2.11, *p* ≥ 0.150), gender will not be examined in the neurophysiological analyses.

### Alpha and theta band power modulations

**Figure 3:**
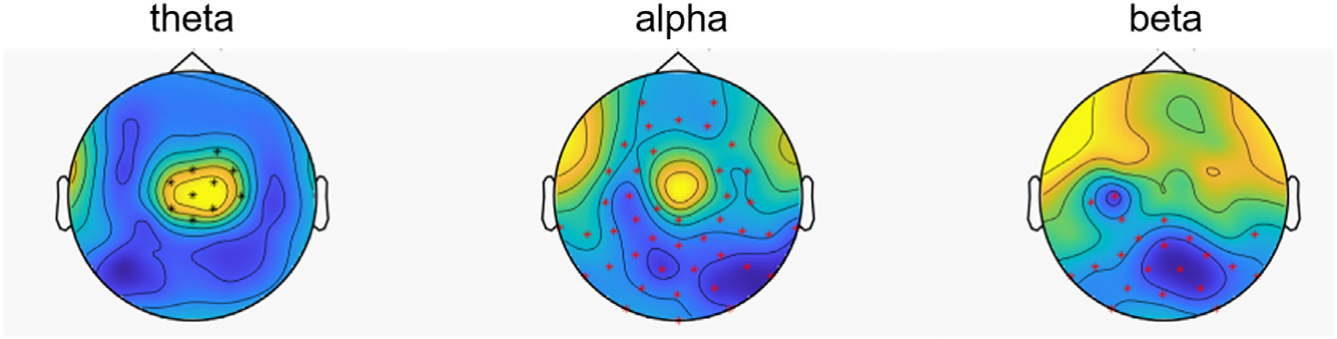
Cluster-based permutation testing Results of the cluster-based permutation testing for theta (left), alpha (center), and beta (right) frequency bands.

To examine in which frequency band differences occur between the non-overlapping and overlapping Nogo condition, cluster-based permutation testing (using data averaged over frequencies within the frequency band and over the time) was applied at the sensor level. The cluster-based permutation testing established significant differences in the theta, alpha, and beta frequency bands between the non-overlapping and the overlapping Nogo conditions (Figure 3). The difference between conditions was calculated by subtracting the non-overlapping from the overlapping condition. Regarding theta band activity (TBA), a positive cluster was found (*p* = 0.006) spanning across fronto-central electrodes (Cz, FCz. FC1, CP1, FC2, CP2, CPz, F2, FC4, and C4), showing that TBA was stronger in the overlapping compared to the non-overlapping condition. With respect to alpha band activity (ABA), a negative cluster was found (*p* = 0.002) spanning across parietal, occipital, and frontal electrodes (CP1, F1, FC3, C3, CP3, P1, AFz, AF3, F5, FC5, C5, CP5, P3, PO1, Fp1, TP7, P7, O1, TP9, P9, O9, P11, CPz, FC4, C4, CP4, P2, Pz, AF4, F6, FC6, C6, CP6, P4, PO2, Fp2, TP8, P8, O2, Oz, TP10, P10, O10, Iz, and P12), showing that ABA was higher in the non-overlapping than in the overlapping condition. The same direction of effect was established for beta band activity (BBA), where a negative cluster was found (*p* = 0.008) encompassing parieto-occipital electrodes (C3, CP3, P1, C5, P3, PO1, P7, O1, P9, O9, CPz, P2, Pz, P4, PO2, TP8, P8, O2, Oz, P10, O10, and P12).

### Source localization of frequency band activity

Since all three frequency bands of interest showed significant power differences at the sensor level, the activity in all three frequency bands was subjected to beamforming analyses with the subsequent clustering of voxels by means of the DBSCAN algorithm. These analyses were conducted separately for both Nogo conditions, i.e., the regions with the highest activity were searched for. With regard to TBA, in both conditions, two clusters were established in the right middle temporal cortex (θ r-MT) and in the left medial occipito-temporal cortex (θ l-MOT). For ABA, three clusters were found in both conditions in the right inferior frontal cortex (α r-IF), the right medial occipito-temporal cortex (α r-MOT), and the right medial occipital cortex (α r-MO). Regarding BBA, in both conditions, only one cluster was established in the medial occipito-temporal cortex (β MOT). The Brodmann area of regions is displayed in Table 1.

**Table 1:** Cluster labels and corresponding Brodmann areas for the three frequency bands

Theta		Alpha		Beta	
Label	Brodmann	Label	Brodmann	Label	Brodmann
θ r-MT	BA21	α r-IF	BA45 BA47	β MOT	BA17 BA18 BA19
θ l-MOT	BA18 BA19	α r-MOT	BA18 BA19	–	–
–	–	α r-MO	BA17	–	–

*Note.* θ r-MT, right middle temporal cortex in theta frequency band; θ l-MOT, left medial occipito-temporal cortex in theta frequency band; α r-IF, right inferior frontal cortex in alpha frequency band; α r-MOT, right medial occipito-temporal cortex in alpha frequency band; α r-MO, right medial occipital cortex in alpha frequency band; β MOT, medial occipito-temporal cortex in beta frequency band; BA, Brodmann area.

### Network analysis for theta and alpha frequency bands (nCREANN results)

**Figure 4:**
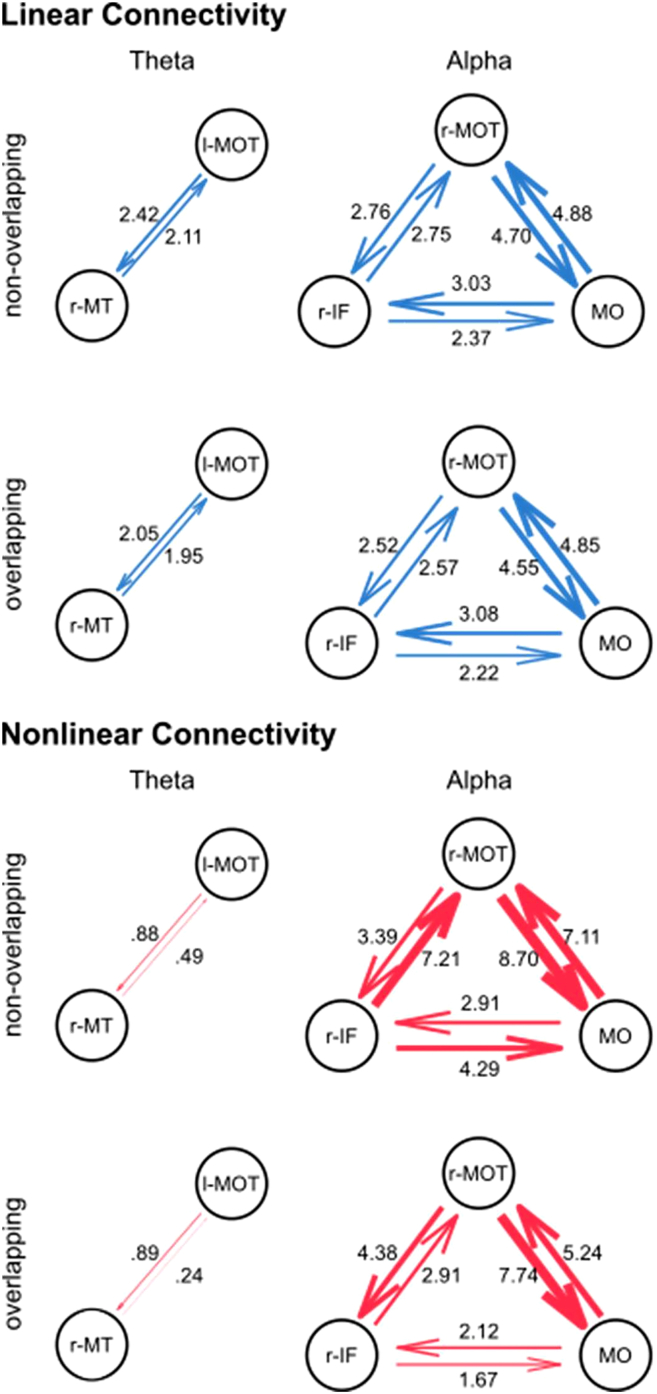
Linear and nonlinear connectivity pattern for theta and alpha frequency bands for non-overlapping (top) and overlapping (bottom) conditions Linear connectivity values are plotted with blue arrows, and non-linear values are plotted with red arrows, showing effects from one region to another. The connectivity values were scaled by a factor of 100 to enhance the clarity of the figure, making them easier to comprehend on the figure.

To evaluate the brain network organization in each frequency band, nCREANN was applied to the time courses of the source signals in theta and alpha frequency bands. Since there was only one dominant cluster in the beta band (Table 1), this frequency band was excluded from the directed connectivity analysis. The model validation measures for the theta and alpha bands for both condition were ${MSE}_{\left(train\right)}\leq 0.023\;\pm 0.007$, ${MSE}_{\left(test\right)}\leq 0.018\;\pm 0.004$ and $R_{\left(train\right)}^{2}\geq 0.993\pm \;0.002$, $R_{\left(test\right)}^{2}\geq 0.997\pm \;0.002$. Figure 4 shows the patterns of average linear (top-blue) and nonlinear (bottom-red) connectivity. The arrow thickness and arrowhead size are proportional to connectivity strength. The average linear connectivity in the alpha band (non-overlapping/α-Mean: 0.034; overlapping/α-Mean: 0.033) is slightly stronger than the theta (non-overlapping/θ-Mean: 0.023; overlapping/θ-Mean: 0.020). For the nonlinear connectivity, there is much stronger average nonlinear connectivity in the alpha band (non-overlapping/α-Mean: 0.056; overlapping/α-Mean: 0.040) rather than the theta band (non-overlapping/θ-Mean: 0.007; overlapping/θ-Mean: 0.006). In addition, both linear and nonlinear relationships are stronger in the non-overlapping condition than in the overlapping one for theta and alpha frequency bands.

**Figure 5:**
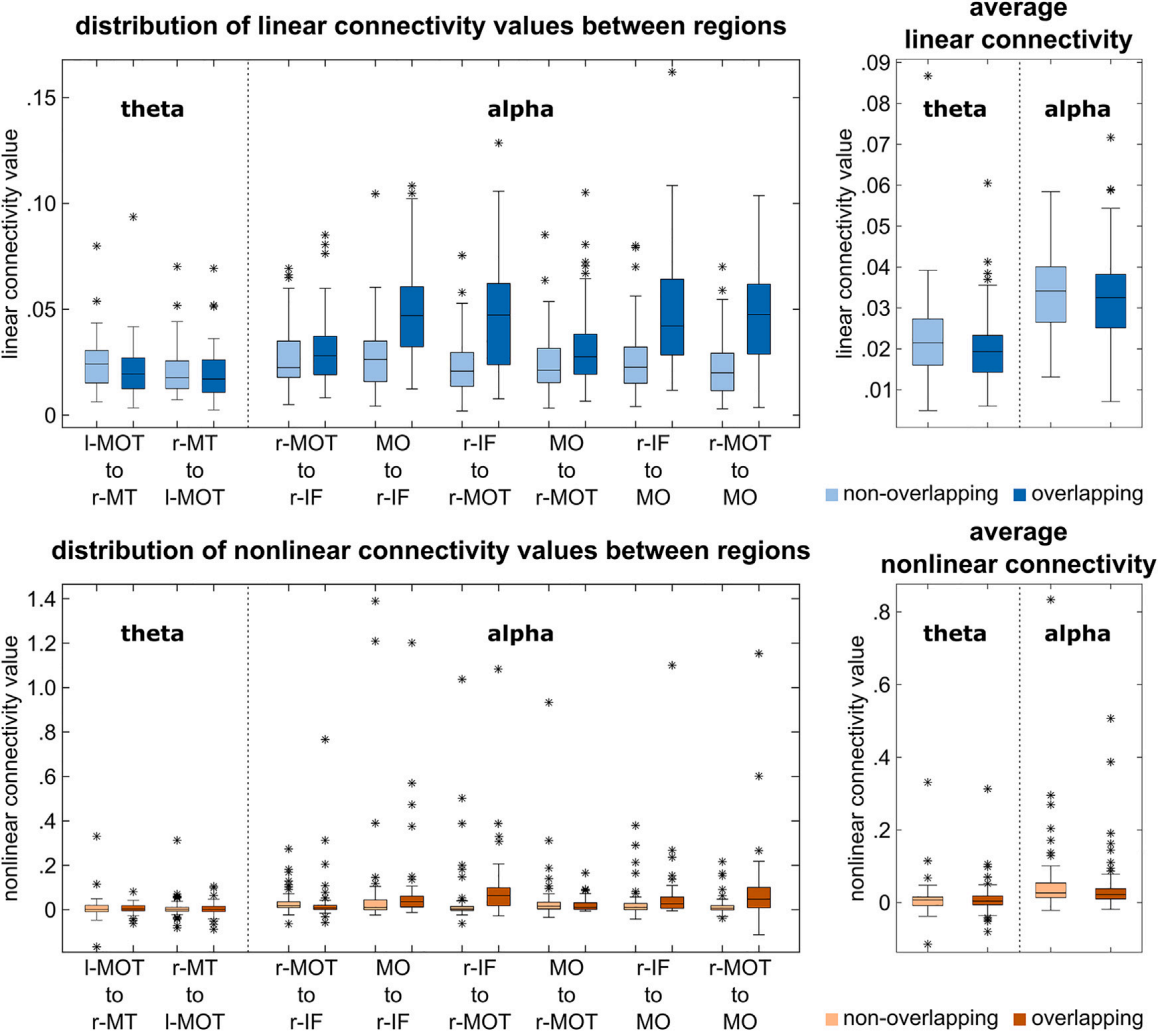
Distribution of nCREANN values in alpha and theta connectivity networks The upper panel shows the distribution of the linear connectivity values separately for the conditions, the lower panel shows the distribution of the nonlinear connectivity values separately for the conditions. The left graph shows the connectivity values separately for each connectivity in the respective network, the right graph shows the average connectivity values in the respective network.

Considering linear connectivity, one-sided t-tests established significant condition differences only for the connectivity from the left medial occipital cortex to the right middle temporal cortex within the theta frequency band (t(59) = 2.21, *p* = 0.016, d = 0.285), with larger connectivity values in the non-overlapping (0.024 ± 0.012) compared to the overlapping condition (0.021 ± 0.011). None of the other linear connectivities within the alpha or the theta frequency band differed significantly between the conditions (t ≤ 1.41, *p* ≥ 0.081). However, no significant correlation could be established between the magnitude of the linear connectivity from the left medial occipital cortex to the right middle temporal cortex within the theta frequency band and the behavioral performance (non-overlapping: r = 0.007, *p* = 0.957; overlapping: r = 0.038, *p* = 0.756). Regarding the nonlinear connectivity, one-sided t-tests showed no significant condition differences neither within the alpha nor the theta frequency band (t ≤ 1.39, *p* ≥ 0.086). The distribution of the connectivity values is represented in Figure 5 (left side).

Considering the average connectivity strength in the alpha and theta connectivity networks, respectively, one-sided t-tests established a significant condition difference only for the average linear connectivity in the theta frequency band (t(75) = 1.94, *p* = 0.028, d = 0.222), with larger connectivity values in the non-overlapping (0.023 ± 0.011) compared to the overlapping condition (0.020 ± 0.009). However, there was no correlation between the linear connectivity values in the theta network and the false alarm rate, i.e., the behavioral performance, neither in the non-overlapping nor on the overlapping condition (r ≤ 1.13, *p* ≥ 0.247). None of the other linear or non-linear connectivities in the alpha or theta connectivity network showed significant condition differences (t ≤ 1.44, *p* ≥ 0.077). The distribution of the average connectivity values is represented in Figure 5 (right side).

### Concomitant network analysis including all frequency bands (nCREANN results)

**Figure 6:**
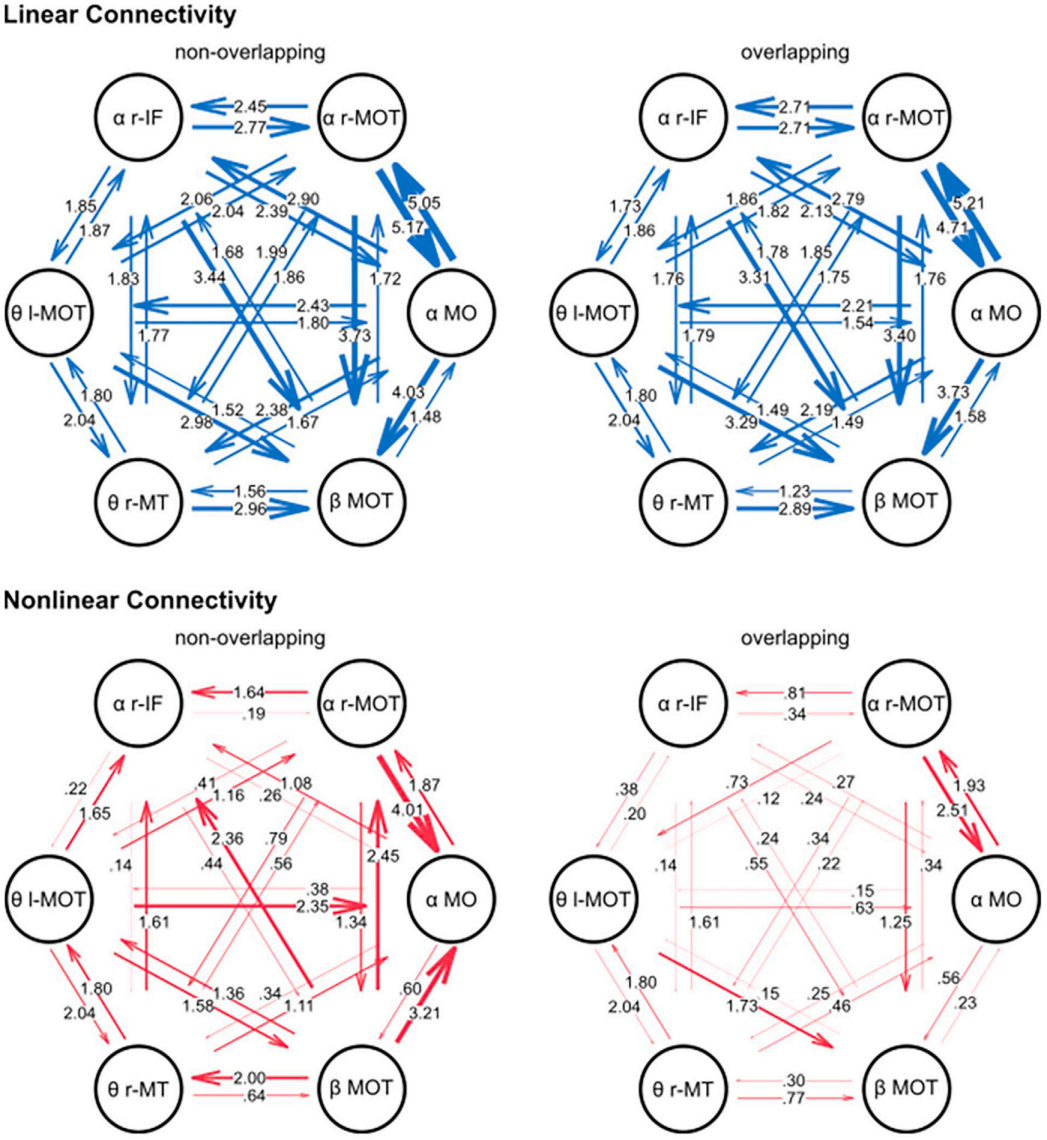
Linear and nonlinear connectivity patterns for all interacting frequencies Active regions of theta, alpha, and beta frequency bands are analyzed simultaneously for non-overlapping (top) and overlapping (bottom) conditions. Linear connectivity (left) values are plotted with blue arrows, and nonlinear values (right) are plotted with red arrows, showing effects from one region to another. The connectivity values were scaled by a factor of 100 to enhance the clarity of the figure, making them easier to comprehend on the figure.

In this analysis, the source clusters of theta, alpha, and beta frequency bands were considered simultaneously for the nCREANN analysis. The model validation measures for the model including all frequency bands were ${MSE}_{\left(train\right)}\leq 0.021\;\pm 0.007$, ${MSE}_{\left(test\right)}\leq 0.028\;\pm 0.008$ and $R_{\left(train\right)}^{2}\geq 0.992\pm \;0.005$, $R_{\left(test\right)}^{2}\geq 0.995\pm \;0.005$ for both condition. Figure 6 shows the patterns of linear (top-blue) and nonlinear (bottom-red) interactions among the regions for non-overlapping and overlapping conditions. The figure indicates that the linear connectivity pattern is almost similar for both conditions. There are strong linear effects from θ r-MT, θ l-MOT, α r-IF, and α -MO to the active region of the beta frequency band, β -MOT.

As it is clear from the figure, in non-overlapping conditions, the active regions of the theta and alpha band have a considerable nonlinear interaction with the active region of the beta band. Furthermore, there is a strong effect from θ r-MT, θ l-MOT, and α r-MOT to α r-IF, and from θ l-MOT, and β -MOT to α -MO. Additionally, there is a significant reciprocal relationship between α -MO and α r-MOT.

**Figure 7:**
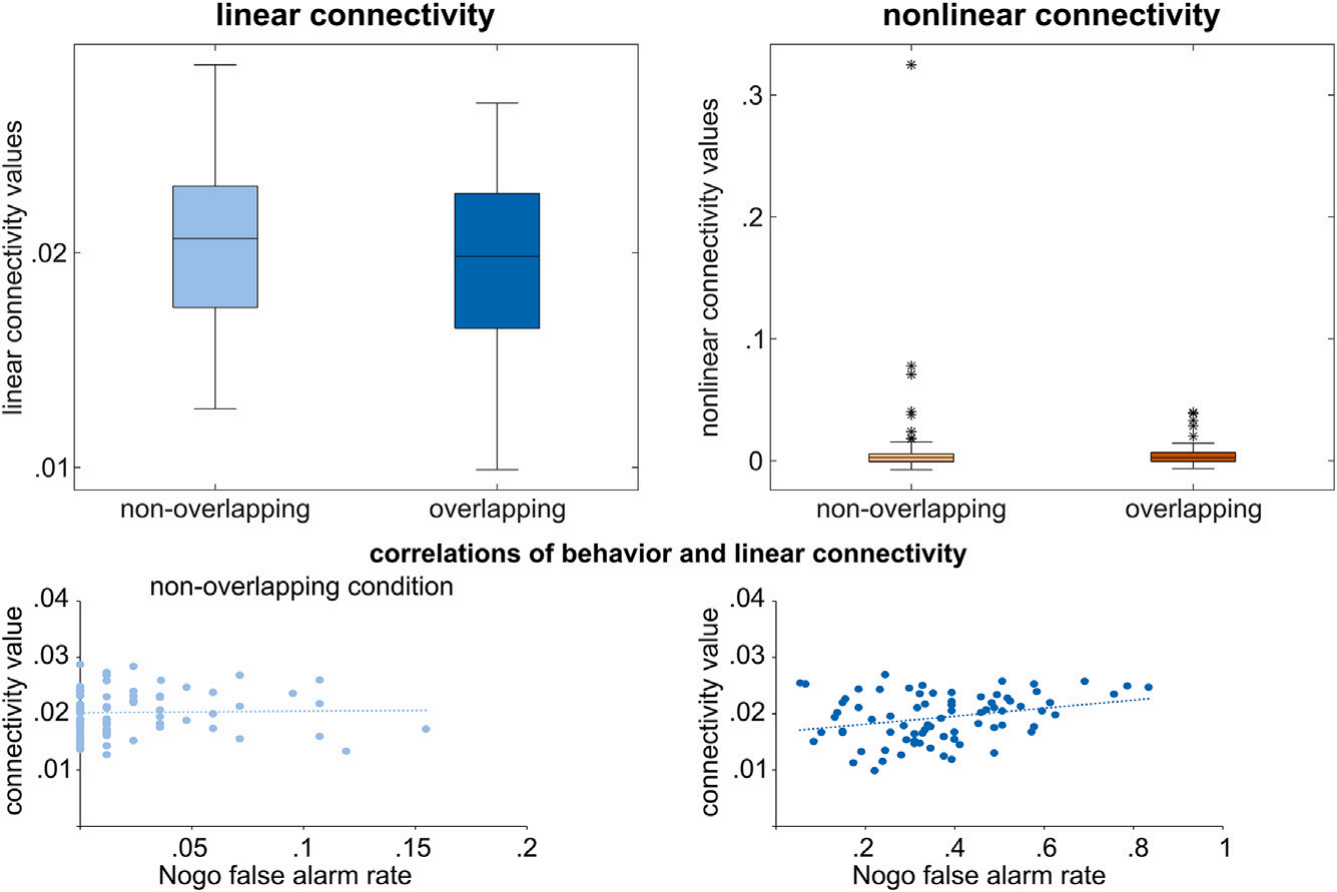
nCREANN values and their correlation with false alarm rates The upper panel shows the distribution of the linear and nonlinear connectivity values separately for the conditions. The scatterplots display the relationship between the linear connectivity values with the false alarm rate in each condition.

Considering the average linear and mean nonlinear connectivity, i.e., the mean of all linear or nonlinear connectivity values, one-sided paired t-tests revealed significant differences between conditions for the average linear connectivity (non-overlapping: 0.025 ± 0.005; overlapping: 0.024 ± 0.005; t(78) = 2.35, *p* = 0.012, d = 0.264), but not for the average nonlinear connectivity (non-overlapping: 0.012 ± 0.046; overlapping: 0.006 ± 0.011; t(78) = 1.16, *p* = 0.125). The distribution of the average connectivity values is represented in Figure 7. In order to associate the significantly different neurophysiological connectivity results and the behavioral performance, a correlation analysis was conducted between the average linear connectivity in each condition and the false alarm rate in the respective condition. There was no significant correlation in the non-overlapping condition (r = 0.026, *p* = 0.822), but a significant positive correlation in the overlapping condition (r = 0.290, *p* = 0.010). The scatterplots of the correlations are presented in Figure 7.

## Discussion

The current study investigated how the directed linear and nonlinear connectivity profile between different functional neuroanatomical regions is modulated during perception-action integration. The behavioral data replicated the pattern found in previous studies: response inhibition performance decreased in overlapping trials compared to non-overlapping trials,^7^^,^^8^^,^^17^^,^^34^^,^^35^^,^^36^ indicating worse inhibitory control when there was a feature overlap necessitating the reconfiguration of event files. Likewise, in Go trials, the hit rate was lower and the reaction times were slower in overlapping than in non-overlapping trials.

The effects on power modulations in the different frequency bands observed at the electrode level are in keeping with previous studies.^8^^,^^17^^,^^36^ For theta band activity (TBA), the activity increased in overlapping compared with non-overlapping trials, indicating an increased need for cognitive control^33^ and more demanding event file reconfiguration processes.^4^ TBA in both Nogo conditions was associated with the right middle temporal gyrus and the left medial occipitotemporal/lingual gyrus, known to encode the used stimulus features colors, and letters.^37^^,^^38^^,^^39^^,^^40^^,^^41^ These stimulus features are decisive in whether to execute or inhibit a response. Therefore, it is reasonable that TBA is high in these areas during the inhibition process.

Concerning alpha band activity (ABA), there was an inversed pattern compared to TBA on the electrode level. ABA has been suggested to reflect inhibitory gating processes regulating whether novel information can update mental representations.^42^ The pattern of an increased ABA in the non-overlapping conditions most likely reflects an increased shielding of information in the event file, for which there is no need to become reconfigured in the non-overlapping condition. In the overlapping condition, such an inhibitory shielding would prevent the necessary reconfiguration and updating of the event file. Therefore, it is necessary that inhibitory gating is reduced, which is reflected by the lower ABA in the overlapping condition. In both conditions, the highest ABA was associated with the right inferior frontal cortex, which is part of a cortical network supporting response inhibition processes.^43^^,^^44^ Moreover, ABA was high in the right medial occipitotemporal/lingual gyrus and the right medial occipital cortex/cuneus, which are involved in lower-level visual processing and attentional processes.^45^^,^^46^ Similar to ABA, there was also increased beta band activity (BBA) in non-overlapping compared to overlapping Nogo trials at the electrode level. Recent concepts relate BBA to the maintenance of event files^4^ which is in line with conceptions of BBA suggesting that it reflects activations of task relevant specific content.^47^ The content of an event file does not have to be reconfigured/altered in the non-overlapping condition. In the overlapping conditions, however, different content has to be activated. Therefore, BBA decreases in the overlapping condition. In both Nogo conditions, BBA was highest in the right medial occipitotemporal/lingual gyrus, which is related to rather global processing of visual input such as words and the discrimination of different stimuli.^46^^,^^48^

Taken together, and also in synopsis with previous studies examining the role of frequency band activity in event file coding in response inhibition,^8^^,^^17^^,^^36^ when there is a need to reconfigure an event file, there are local increases of TBA reflecting a need for control processes in regions processing relevant stimulus information to guide response selection. The concomitant necessity to enable an updating of the event file (i.e., reduce shielding against different information) and the fact that novel content-specific information has to be implemented in the event file, ABA and BBA decrease in regions processing relevant stimulus information to guide response selection, respectively. This is in keeping with findings, according to which the involvement of cortical regions depends on the *identity* of features constituting a stimulus that is bound to a specific response.^11^ Importantly, this pattern of local activity changes has to be put in context with modulations of directed information transfer.

Considering the network encompassing all frequency bands, there is a decrease in linear connectivity strength in the overlapping compared with the non-overlapping condition. This finding is in line with the predictions of TEC that the reconfiguration of an event file necessary in the overlapping condition requires weakened connections within a network in order to integrate new information.^6^ However, only considering both the local activity and the connectivity between the involved areas separately in the different frequency bands allows for more detailed insights.

In the TBA network, directed linear connectivity decreases in the overlapping condition, compared to the non-overlapping condition. Thus, there is a dissociation of local activity increases, as indicated by the established differences on the sensor level and previous findings of modulations in frontal and temporal areas,^8^^,^^17^^,^^36^ and directed connectivity decreases within theta band dynamics. This decrease of directed communication seems to be particularly relevant when it comes to the directed transfer of information from lower-order visual areas (i.e., the medial occipitotemporal/lingual gyrus) to higher-order visual areas (i.e., the middle temporal gyrus). The dissociation of local activity and directed transfer of information between cortical regions in the theta band stresses the unique role of this frequency band for perception-action integration. According to the TEC framework and its derivatives,^2^^,^^4^ event file dynamics reflect network dynamics obeying a pattern-completion logic.^32^ Low-frequency/high-amplitude oscillations – such as TBA – are optimal for the integration of information processed in distant brain regions,^49^ which is likely the case in event files. The integration of information into an event file and the retrieval of it are most likely determined by theta band dynamic.^4^ During event file retrieval, reconfiguration processes can become necessary (see above). The observed theta band dynamics likely reflect a means to comply with two simultaneous requirements: (i) the need to increase control during event file reconfiguration in cortical structures processing relevant information to enable behavioral adaptation (local TBA; see above) and (ii) with the necessity to weaken directed connectivity between involved brain areas to facilitate the integration of relevant new information into an event file during the reconfiguration processes.

Taken together with the findings considering the network encompassing all frequency bands, this suggests that it is the joined effect of TBA, ABA, and BBA directed linear information transfer between cortical regions that enable the dynamic reconfiguration of event files and behavioral adaptation. This interplay between the localization of processes in the brain and the temporal unfolding of brain processes as well as the temporal influence of one brain region on another is also in line with the recent approach of spatiotemporal neuroscience, which suggests that it is the *where* and *when* of brain activity that underlies cognitive functioning.^19^^,^^20^ Crucially, this interpretation is supported by the results of the correlation analyses: only in situations requiring event file reconfiguration, lower average linear connectivity strength was associated with lower false alarm rates, i.e., better performance. Importantly, the correlation was obtained for the connectivity strength of the network in which TBA, ABA, and BBA were considered together only, which directly shows that network dynamics in all frequency bands is important to consider for perception-action integration. In fact, it has been proposed that TBA, ABA, and BBA serve distinct, but interconnected subprocesses during the management of perception-action integration.^4^ The obtained findings directly support this conception.

### Limitations of the study

Although the current study already offers valuable insights into the underlying sources of frequency band activity, the precision of this source localization might be enhanced in future studies by using more electrodes for the measurement. Furthermore, while the current study investigates oscillatory activity as well as directed connectivity measures between brain regions, future research on the causal effective interactions between brain regions within and between different frequency bands needs to be conducted to gain deeper insights into the causal relationships.

### Conclusions

In summary, the flexible management of perceptual and motor processes depends on the close interplay of the alpha, beta, and theta frequency bands. Importantly, directed linear connectivity within a temporo-occipital theta band network becomes concomitantly weaker if perception-action associations have to be modified, whereas local theta band activity increases when such a modification is necessary. Moreover, the interplay of alpha, beta, and theta frequency bands explained behavioral data, showing that a weaker connectivity led to a better inhibition performance when the reconfiguration of perception-action associations was required. Thus, adaptive behavior relies on concomitantly occurring and partly opposing processes: (i) local modulations of activity in frontal and sensory cortices depending on the frequency band, and (ii) concomitant decreases in directed connectivity between these regions. The findings reframe concepts on neural processes supporting adaptive behavior.

## Resource availability

### Lead contact

Further information and requests for resources and reagents should be directed to and will be fulfilled by the lead contact, Christian Beste (Christian.beste@ukdd.de).

### Materials availability

This study did not generate new unique reagents or other materials.

### Data and code availability

• Behavioral and EEG raw data have been deposited at the open science framework (OSF) and are publicly available at https://osf.io/6vn3f/.
• All original code has been deposited at OSF and is publicly available at https://osf.io/6vn3f/.
• Any additional information required to reanalyze the data reported in this article is available from the lead contact upon request.

## Acknowledgments

This work was supported by Grants from the 10.13039/501100001659Deutsche Forschungsgemeinschaft (DFG) FOR 2790 and FOR 2698 and by a Grant from the 10.13039/501100007148Bundesministerium für Wissenschaft und Forschung (10.13039/501100002347BMBF) 13GW0557B.

## Author contributions

Conceptualization: C.B.; formal analysis: N.T. and A.P.; funding acquisition: C.B., C.F., and A.M..; investigation: A.P.; methodology: N.T., A.P., and C.B.; project administration: C.B.; resources: C.B.; software: N.T. and M.M.; supervision: C.B.; validation: N.T. and A.P.; visualization: N.T. and A.P.; writing – original draft and preparation: N.T., A.P., and C.B.; writing – review & editing: all authors. All of the authors reviewed and approved the article for publication.

## Declaration of interests

The authors declare no competing interests.

## STAR★Methods

### Key resources table

**Table:** 

REAGENT or RESOURCE	SOURCE	IDENTIFIER
**Deposited data**		

Raw data behavior	This paper	https://osf.io/6vn3f/
Raw data EEG	This paper	https://osf.io/6vn3f/

**Software and algorithms**		

MATLAB	https://de.mathworks.com/products/matlab.html	RRID:SCR_001622
BrainVision Recorder	https://www.brainproducts.com/productdetails.php?id=21	RRID:SCR_016331
EEGLAB	http://sccn.ucsd.edu/eeglab/index.html	RRID:SCR_007292
FieldTrip	https://www.fieldtriptoolbox.org	RRID:SCR_004849
Presentation	http://www.neurobs.com/	RRID:SCR_002521
SPSS	http://www-01.ibm.com/software/uk/analytics/spss/	RRID:SCR_002865

### Experimental model and study participant details

#### Sample

We assessed *N* = 91 healthy participants aged 20 to 30 years. Participants were recruited as a convenience sample via advertisements and an in-house database. After exclusions due to not meeting inclusion criteria (*N* = 8), problems understanding the instruction (*N* = 1), or technical problems (*N* = 3), the final sample size was *N* = 79 subjects (46 males; age: 24.1 ± 2.8 years; IQ: 111 ± 13). The subjects were not further allocated to experimental groups. There is a partial overlap of this sample with previous studies of our workgroup investigating the same task.^7^^,^^8^^,^^17^^,^^34^^,^^35^^,^^36^ All included participants reported the absence of psychiatric or neurological disorders during a brief telephone screening, which was underpinned by the *Adult Self-Report* (ASR/18–59)^50^ and the *Alcohol, Smoking and Substance Involvement Screening Test* (ASSIST).^51^ Before study procedures started, the subjects provided written informed consent. They received a financial compensation or course credits for their participation. The local ethics committee of the TU Dresden approved the study.

### Method details

#### Task

Perception-action integration in the context of response inhibition was investigated by having participants performing a Go/Nogo task modified on the basis of the TEC.^9^ The stimuli and the course of a trial are displayed in Figure 1.

As can be seen in Figure 7, The Go and Nogo stimuli in this task had the features "color" and "letter sequence", which could either be completely distinct between Go and Nogo stimuli (non-overlapping condition) or were overlapping between Go and Nogo stimuli (overlapping condition). This resulted in the following stimuli: In the non-overlapping Go condition, subjects saw a green-colored "PRESS", whereas in the non-overlapping Nogo condition they saw a red-colored "STOPP". The overlapping Go conditions were either a white-colored "DRÜCK" or a blue-colored "XXXXX". In the overlapping Nogo condition, either a blue-colored "DRÜCK" or a white-colored "XXXXX" was presented. Subjects were instructed to respond to Go stimuli by pressing the space bar, whereas they were instructed to not press any key when presented with Nogo stimuli. Thus, since Go stimuli required a response, but Nogo stimuli required inhibition, the overlapping of stimulus features in the overlapping condition resulted in an overlap of event files, which in particular should induce higher false alarm rates in terms of partial-overlap costs. Before performing the task, subjects completed practice trials to familiarize themselves with the task. In the task itself, there were 196 trials in each Go condition (green "PRESS," white "DRÜCK," blue "XXXXX") and 84 trials in each Nogo condition (red "STOPP," blue "DRÜCK," white "XXXXX"). This resulted in a 70:30 ratio of Go to Nogo trials, which aimed to establish a prepotent response tendency.^52^ Trials were divided into seven blocks of equal length, in which each condition was presented equally often in pseudorandomized order. Between blocks, subjects could take a short break of self-chosen length. Each trial began with the presentation of the stimulus for 450 ms and ended either with a key press by the subject or after 1700 ms if no key was pressed. Between trials, the inter-trial interval was jittered between 700 and 1100 ms. When no stimulus was shown, a white fixation cross was displayed in the center of the screen.

For all further analyses the overlapping Go conditions (i.e., white “DRÜCK” and blue “XXXXX”) were combined. Also, the overlapping Nogo conditions (i.e., blue “DRÜCK” and white “XXXXX”) were merged for all further analyses, including the neurophysiological analyses. For the analyses of the behavioral data, the false alarm rates, the Go hit rates, and the Go reaction times were averaged over the different overlapping and non-overlapping trials. For the neurophysiological analyses, only Nogo trials were considered, as only these reflect the response inhibition being of interest. Comparisons between the overlapping and non-overlapping conditions were performed using paired t-tests and Cohen’s d was calculated to estimate the effect size.

#### EEG recording and pre-processing

The EEG signals were recorded using a BrainAmp amplifier (BrainProducts Inc.) from 60 Ag/AgCl electrodes, which were equidistantly positioned in an elastic cap (EasyCap M10). The reference electrode was located at Fpz, the ground electrode was located at θ = 58, ϕ = 78, impedances were kept below 5 kΩ. As a first step of offline-pre-processing, the data were down-sampled to 256 Hz. For the pre-processing, the “Automagic” pipeline^53^ and EEGLAB^54^ on MATLAB were applied. First, EOG channels (FP1, AF7, FP2 and AF8) were checked and removed if they were flat and the data were re-referenced to an average reference afterward. Subsequently, the PREP preparation pipeline^55^ and the EEGLAB “clean rawdata()” pipeline were implemented: The PREP pipeline employs a multitaper algorithm for the removal of line noise (50 Hz), removes contamination from the dataset by removing bad channels, and afterward adds a robust average reference. The “clean rawdata()” pipeline, on the other hand, applies a 0.5 Hz FIR high-pass filter (order 1286, stop-band attenuation −80 dB, transition band 0.25–0.75 Hz) for the detrending of the EEG signals, whereby noisy or flat-lined channels and outliers were identified and removed. For the reconstruction of epochs with abnormally strong power (>15 standard deviations relative to calibration data) in the segmented data (see below), Artifact Subspace Reconstruction (ASR; burst criterion;^56^) was applied. Time periods that could not be reconstructed were removed. Afterward, a low-pass filter of 40 Hz (sinc FIR filter; order: 86;^57^) was applied to the data. In the next step, EOG artifacts were removed using a subtraction method,^58^ whereas other periodic artifacts (muscle, heart, remaining eye artifacts) were automatically organized and eliminated by applying an Independent Component Analysis (ICA) based on the Multiple Artifact Rejection Algorithm (MARA;^59^^,^^60^). The average number and std of removed channels and removed ICs across all subjects were 5.392 ± 3.027 and 17.430 ± 4.856, respectively. The interpolation of absent and removed channels was done using a spherical method. The data segmentation into 4000 ms (−2000 ms–2000 ms relative to stimulus onset) segments was performed using the BrainVision Analyzer 2.0 (BrainProducts Inc.).

#### Time-frequency analysis

Time-frequency (TF) analyses were conducted, using FieldTrip.^61^ The Morlet wavelets technique with a width of five cycles was applied to estimate time-frequency information of power spectrum. In order to have a sufficient number of cycles for the analyses in the frequency bands, the length of the intervals to be analyzed was set at 4000 ms (from −2000 to 2000 ms after stimulus onset). The average power was calculated for each time point and electrode separately in the theta (4–7 Hz), alpha (8–12 Hz), and beta (12–25 Hz) frequency bands, respectively.

In order to analyze the differences between non-overlapping and overlapping Nogo trials in terms of electrode activity, cluster-based permutation tests were conducted for each frequency band within the time interval of −1000 to 1000 ms, using FieldTrip. Electrodes belonging to a cluster were determined by a paired sample t-test with a *p*-value below 0.05 (two-tailed) and at least two adjacent EEG channels. The reference distribution was obtained by performing 500 random draws using the Monte Carlo method. For each subject, the wavelet-based time-frequency information is computed for each single trial, and then it is averaged over all trials (each condition separately). We then set the parameter to average the power over −1000 ms to +1000 ms and then run the statistics for the specified frequency bands.

Furthermore, the time series of EEG signals in theta, alpha, and beta frequency bands were extracted using Hamming windowed sinc FIR filters. The filtered signals were then utilized for beamforming and connectivity analysis.

#### Beamforming analysis

For the source localization and the subsequent connectivity analysis using nCREANN method (see below), the Nogo conditions (non-overlapping and overlapping) were analyzed separately. To this end, first the sources of activity in each condition were localized using beamforming analyses. Afterward, the directed connectivity was computed separately for each condition. In a final step, the connectivity values were compared statistically between the Nogo conditions.

For estimating directed connectivity from EEG signals it is critical to consider the volume conduction effect; the electrical activity’s transmission from the brain through conductive tissues (such as the scalp and skull) and fluids (such as cerebrospinal fluid) to the electrodes positioned on the scalp. This effect can confound an accurate estimation of connectivity.^62^^,^^63^ Assessment of the connectivity of the underlying sources reconstructed from EEG recordings is a frequent strategy to this problem. In the current study, similar to previous studies,^17^^,^^64^ the source time courses in the mentioned frequency bands within each condition was estimated by the linearly constrained minimum variance (LCMV) beamforming technique using FieldTrip.^65^ First, using a template head model, a common spatial filter was calculated from the concatenated time-locked average trials of each non-overlapping and overlapping conditions (the trials of each condition were averaged separately, then the two averaged signals were concatenated). Then, the time courses of the single trial source activities of each condition were computed based on the common spatial filter.

The clusters of theta, alpha and beta activity in the LCMV-beamformed data were then defined by applying the Density-Based Spatial Clustering of Applications with Noise (DBSCAN) algorithm^66^ onto the Neural Activity Index (NAI) of the sources.

The voxel selection was limited to functional neuroanatomical regions with high activity. The threshold was set to the top 1% of the NAI distribution within labeled regions in the Automatic Anatomical Labeling (AAL) atlas.^67^ The minimum cluster size was set to two voxels, with an epsilon value of 1.5 times the edge length of each voxel to detect neighboring voxels. Selected clusters were inspected visually (to exclude too small clusters that could not be deemed meaningful and/or to merge clusters based on neuroanatomical considerations so that they formed neuroanatomically meaningful larger clusters) and chosen for further analysis based on voxel count and anatomical labels. An average of all voxel time courses within each selected cluster was considered as the estimate of activity for that cluster in the connectivity analysis.

#### Directed functional connectivity analysis

To evaluate the directed connectivity patterns in theta, alpha, and beta frequency bands between the clusters established by the DBSCAN algorithm we used the nCREANN (nonlinear Causal Relationship Estimation by Artificial Neural Network) method.^30^^,^^68^ The nCREANN uses an artificial neural network (ANN) to estimate directed connectivity among multiple regions based on a nonlinear Multivariate Autoregressive (nMVAR) model. In an nMVAR model, the current samples of brain regions are generated based on interactions between previous regions. Typically, the nMVAR model is used to represent temporal causality, in which the cause impacts the future. The nCREANN approach captures both linear and nonlinear dynamics of the information flow among cortical areas, in contrast to standard linear methods that only focus on linear MVAR models. Since the complex nonlinear behaviors of the nervous system have been reported from a single neuron to the system level,^69^ linear methods run the risk of oversimplifying the intricate dynamics of brain function. In fact, nonlinear interactions are important for the organisation of information flow across cortical regions.^70^^,^^71^ The relevance of linear and nonlinear principles for a deeper comprehension of neuro-dynamics at macroscale levels has been suggested by several lines of evidence.^72^^,^^73^^,^^74^^,^^75^^,^^76^ A nonlinear MVAR model is defined as:

(Equation 1) $$\left[\begin{array}{c} x_{1}\left(n\right)\\ \vdots \\ x_{M}\left(n\right) \end{array}\right]=\left[\begin{array}{c} f_{1}\left(x_{p}\right)\\ \vdots \\ f_{M}\left(x_{p}\right) \end{array}\right]+\left[\begin{array}{c} \sigma_{1}\left(n\right)\\ \vdots \\ \sigma_{M}\left(n\right) \end{array}\right]$$

Where $x\left(n\right)={\left[x_{1}\left(n\right),x_{2}\left(n\right),\ldots ,x_{M}\left(n\right)\right]}^{T}\in R^{M}$ is the present samples of (M) multivariate time courses of the cluster sources, $x_{p}={\left[x_{1}\left(n-1\right),x_{2}\left(n-1\right),\cdots ,x_{M}\left(n-p\right)\;\right]}^{T}$ is the vector of their p previous samples, and $\sigma \left(n\right)={\left[\sigma_{1},\sigma_{2},\ldots ,\sigma_{M}\;\right]}^{T}$ is the model residual. The functions $f\left(.\right)={\left[f_{1},f_{2},\ldots ,f_{M}\;\right]}^{T}$ represent how the p previous samples generate the present values (Equation 1).

In the nCREANN, a single-hidden-layer feedforward network with nonlinear activation functions for hidden neurons and linear ones at the output layer implements this model. During the training, the present samples at the network’s outputs are predicted based on the interactions among past samples of all the signals. To extract linear and nonlinear directed connectivity from the network, the linear and nonlinear parts of f(.) are segregated using the Taylor expansion of the hidden neurons’ activation function. The Taylor expansion approximates functions by summing linear and nonlinear terms with high accuracy.^77^ Using the Taylor expansion, the functions f can be re-written to

(Equation 2) $$f=f^{Lin}+f^{NonLin}$$

And Equation 1 becomes:

(Equation 3) $$\left[\begin{array}{c} x_{1}\left(n\right)\\ \vdots \\ x_{M}\left(n\right) \end{array}\right]=\sum_{d=\overset{`}{1}}^{p}A_{d}\left[\begin{array}{c} x_{1}\left(n-d\right)\\ \vdots \\ x_{M}\left(n-d\right) \end{array}\right]+\left[\begin{array}{c} f_{1}^{NonLin}\left(x_{p}\right)\\ \vdots \\ f_{M}^{NonLin}\left(x_{p}\right) \end{array}\right]+\left[\begin{array}{c} \sigma_{1}\left(n\right)\\ \vdots \\ \sigma_{M}\left(n\right) \end{array}\right]$$

The first term of the right-hand side of the above equation is the linear part of input-output mapping, $f^{Lin}$. The coefficient $a_{ij}^{d}$ in the matrix $A_{d}$ exclusively represents the linear contribution of $x_{j}\left(n-d\right)$ to $x_{i}\left(n\right)$.

(Equation 4) $$A_{d}=\left[\begin{array}{ccc} a_{11}^{d} & \ldots & a_{1M}^{d}\\ \vdots & \ddots & \vdots \\ a_{M1}^{d} & \cdots & a_{MM}^{d} \end{array}\right],d=1,2,\ldots ,p$$

On the other hand, the details of nonlinear interactions of the signals are represented in the nonlinear part, $f^{NonLin}$ which includes higher-degree polynomial terms of the Taylor series.

##### Linear directed connectivity

*Linear Connectivity*$\left({lC}_{i\to j}\right)$ is considered as the linear impact of *i*^th^ cluster on the *j*^th^ cluster, and is defined as the mean absolute values of $a_{ji}^{d}$ for d=1,2,…,p, (see more information in^31^):

(Equation 5) $${lC}_{i\to j}=\frac{1}{p}\sum_{d=\overset{`}{1}}^{p}a_{ji}^{d}$$

##### Nonlinear directed connectivity

*Nonlinear Connectivity*, $\left({NC}_{i\to j}\right)$, establishes the extent of the nonlinear causal effect of $x_{i}$ on $x_{j}$. It is determined by the ratio of the network’s estimation errors (difference between original and predicted values):

(Equation 6) $${NC}_{i\to j}=\ln\left(\frac{\left\langle {{\left(\epsilon_{j}\right)}_{x_{i}\_Lin}}^{2}\right\rangle }{\left\langle {\left(\epsilon_{j}\right)}^{2}\right\rangle }\right)$$

The error value at the numerator occurs when, $x_{i}$ has only a linear influence $x_{j}$, but other remaining input signals have both linear and nonlinear effect on $x_{j}$. The estimation error in the denominator is for the case that all the input signals have both linear and nonlinear influence on $x_{j}$.

In the present study, the nCREANN was applied to the time courses of the LCMV-derived sources in non-overlapping and overlapping conditions. The data points of the trials in the time interval from −1000 to 1000 ms were considered for the connectivity analysis. All of the trials were concatenated in order to create a dataset with a sufficient length for training the network. Akaike and Schwartz criteria were used to assess the best model order (p=10). p was considered the same for both conditions. A single hidden layer Perceptron neural network with 10 hidden neurons was trained. Putting $x_{p}$ at the network’s input, it tries to predict x(n) as its output. The training algorithm was gradient descent error back-propagation (EBP) with momentum (α) and adaptive learning rate (η). For sake of generalisation, the early stopping technique was applied. The 10-fold permuted cross validation technique was applied and in each fold the data was divided into 80% training, 10% validation, and 10% testing sets. The network parameters were updated in the ‘incremental’ mode (each time an input is presented to the network), with random initial parameters in the range of [-0.5,0.5].

Model validation was determined using Mean Square Error (MSE) and R^2^ on both training and test data. R^2^ is a statistical metric used in regression models to assess their goodness of fit (values close to 1 indicate that the model fits the data well). MSE is the most widely used metric to assess a network’s performance. A well-trained network has both small training and test error. Additionally, the similar R2 values for training and test set emphasize the network’s proper generalisation. Furthermore, the significance of the connectivity estimates was evaluated through generation of 100 surrogate data with time-shifted surrogate method. This technique eliminates any causal relationship between the signals without modifying the internal dynamics of each time series.^78^^,^^79^ The network configurations were similar to the original data for application of nCREANN to the surrogate data. Only single-subject connectivities which were significant according the surrogate test were used for the subsequent analyses.

The connectivity patterns were displayed with the arrows showing the information flow from one cluster to another one. The coordinates of the source clusters were achieved with the DBSCAN analysis (beamforming analysis section) and are presented in the supplemental information (Figures S1–S6). The thickness of the arrows and the arrowhead size were proportional to the connectivity values. The non-self-connections were plotted for the average values across all subjects. The connectivity patterns were shown for the values above a threshold. This threshold was the average of all connections in all frequency bands in both conditions.

To investigate the connectivity patterns in the overlapping and non-overlapping contexts, two scenarios were taken into consideration. First, the linear and nonlinear information flow was evaluated in each individual frequency band. To do this, nCREANN was applied to the source activity in theta and alpha bands (beta band was excluded from the connectivity analysis, since there was only one source cluster (Table 1)). The second scenario was accomplished by including the source clusters of all frequency bands altogether and performing the nCREANN for these clusters as a single system. In other words, the interaction of brain regions in each of theta, alpha and beta bands generate their current activity.

### Quantification and statistical analysis

The behavioral data were analyzed using IBM SPSS (Version 29), while the analysis of the neurophysiological data was conducted using MATLAB and FieldTrip,^61^ as reported above. For the behavioral data analysis and the evaluation of the nCREANN values, the values in the conditions (non-overlapping vs. overlapping condition) were compared using dependent t-tests as implemented in SPSS and MATLAB, respectively. Moreover, to test for the influence of gender on the obtained effects, a mixed effects ANOVA with the within-subject factor *Overlap* (non-overlapping vs. overlapping condition) and the between-subjects factor *Gender* (male vs. female) was conducted in IBM SPSS (Version 29). The sample size is reported above (experimental model and study participant details, Sample), and the degrees of freedom are reported alongside the results in the results section (for t-tests, df=N−1, where N is the final sample size; for ANOVAs, ${df}_{1}=k-1$ and ${df}_{2}=N-k$, where k is the number of groups and N is the final sample size). The means and standard deviations are reported in the results section as well. Results were considered as significant if the respective *p*-value was below 0.05. For the comparison of the nCREANN values, the t-tests were one-sided. For all significant comparisons, Cohen’s d was calculated as effect size. Statistical values displayed in the figures (e.g., definition of center and dispersion measures) are explained in the respective figure legends.

Pearson’s correlation coefficient between connectivity values significantly differing between the conditions and the behavioral performance in Nogo trials, i.e., false alarm rates, was calculated for each condition using MATLAB for the full final sample (*N* = 79 subjects). Results were considered as significant if the respective p-value was below .05.
