# Supplementary material for: Neural mechanisms of adaptive behavior: Dissociating local cortical modulations and interregional communication patterns
Source: iScience. 2024 Sep 20;27(10):110995. doi: 10.1016/j.isci.2024.110995 (PMC11615187; doi:10.1016/j.isci.2024.110995)
Supplement: Document S1. Figures S1–S6 [file mmc1.pdf]

iScience, Volume *27*

Supplemental information

Neural mechanisms of adaptive behavior: Dissociating local cortical modulations and interregional communication patterns

Nasibeh Talebi, Astrid Prochnow, Christian Frings, Alexander Münchau, Moritz Mückschel, and Christian Beste

Supplemental Figures

The Beamforming/DBSCAN-derived clusters were plotted on orthogonal MRI slices of a typical head for non-overlapping and overlapping conditions at theta, alpha, and beta frequency bands. Clusters were shown in different colors. These figure refer to Figures 3 and 5 in the main paper showing directed connectivity patterns between these regions in an abstracted form.

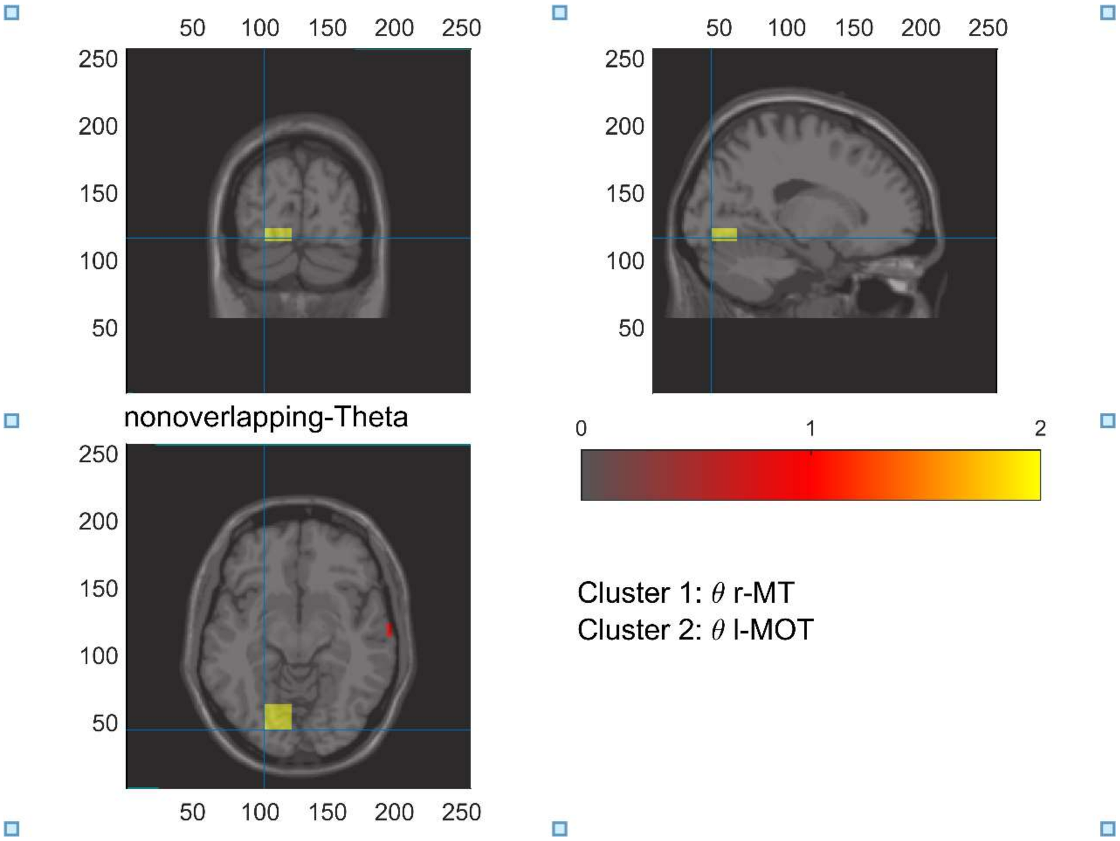

Supplemental Figure S1: Cluster position for the non-overlapping condition at theta frequency band. Related to STAR Methods as well as Figures 3 and 5. Colors indicate the obtained clusters listed below the color bar.

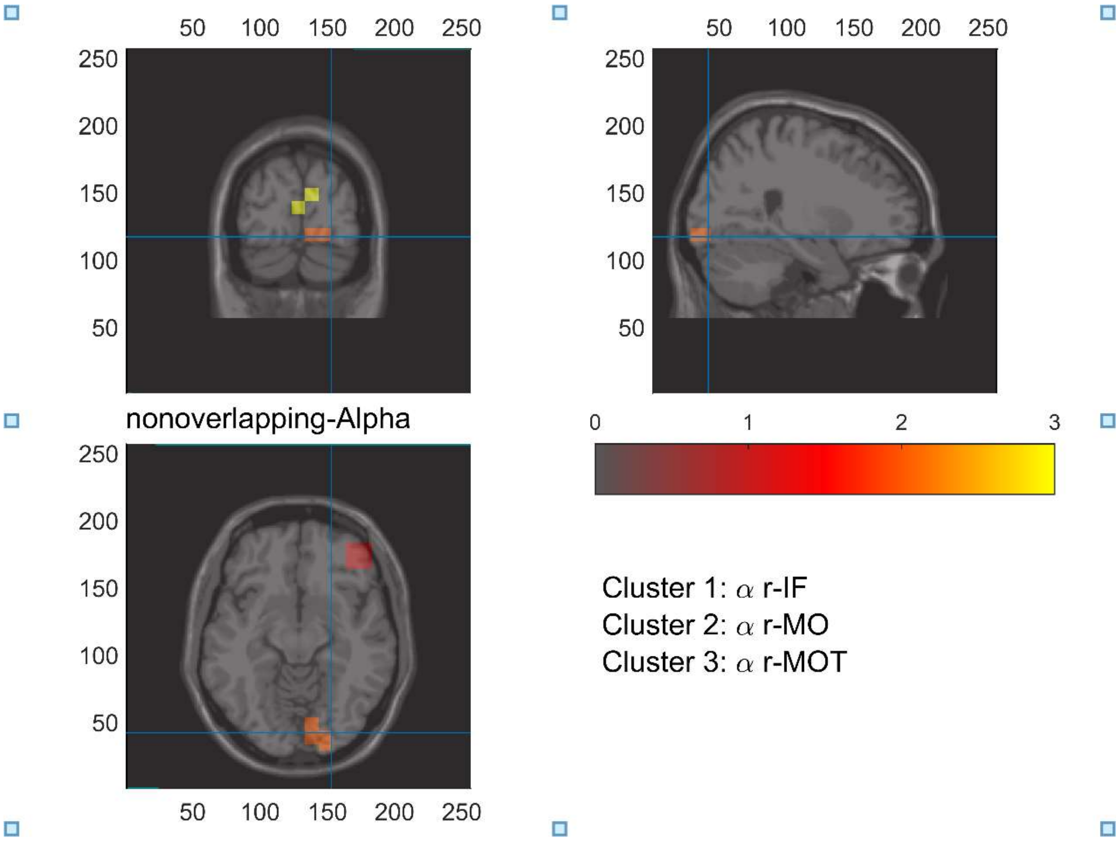

Supplemental Figure S2: Cluster position for the non-overlapping condition at alpha frequency band. Related to STAR Methods as well as Figures 3 and 5. Colors indicate the obtained clusters listed below the color bar.

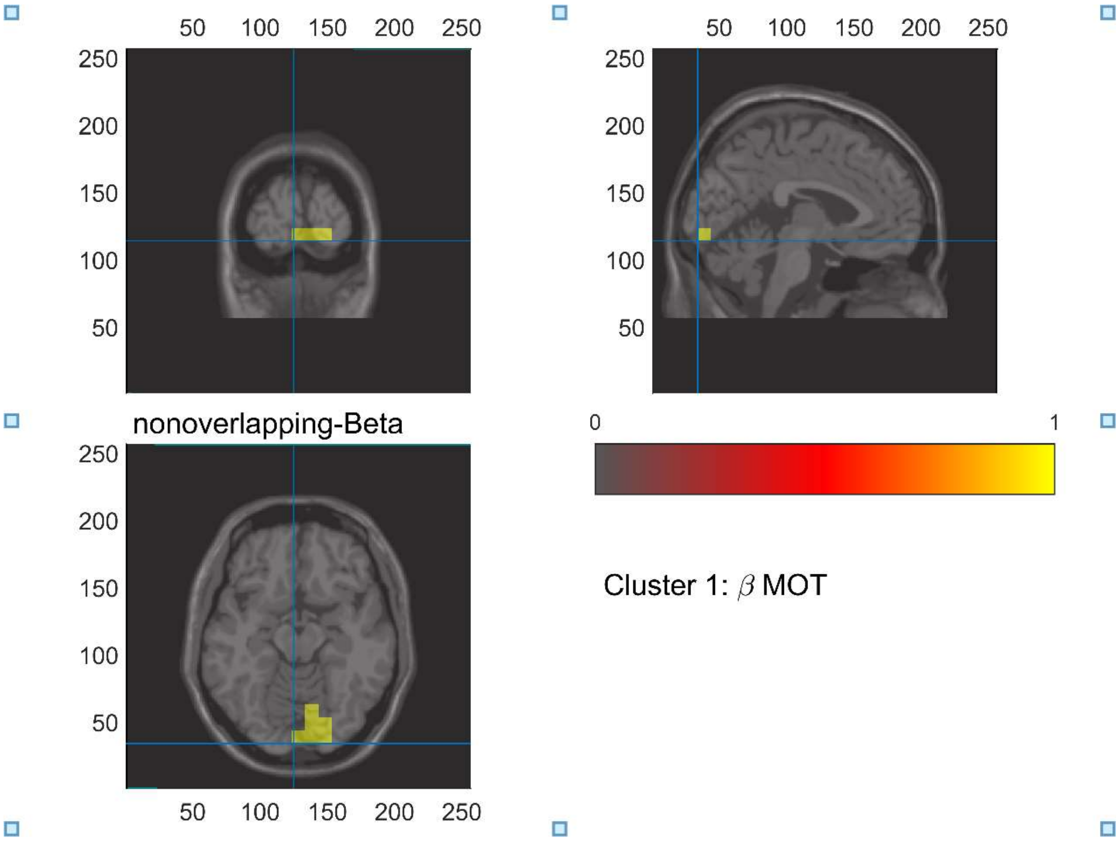

Supplemental Figure S3: Cluster position for the non-overlapping condition at beta frequency band. Related to STAR Methods as well as Figures 3 and 5. Colors indicate the obtained clusters listed below the color bar.

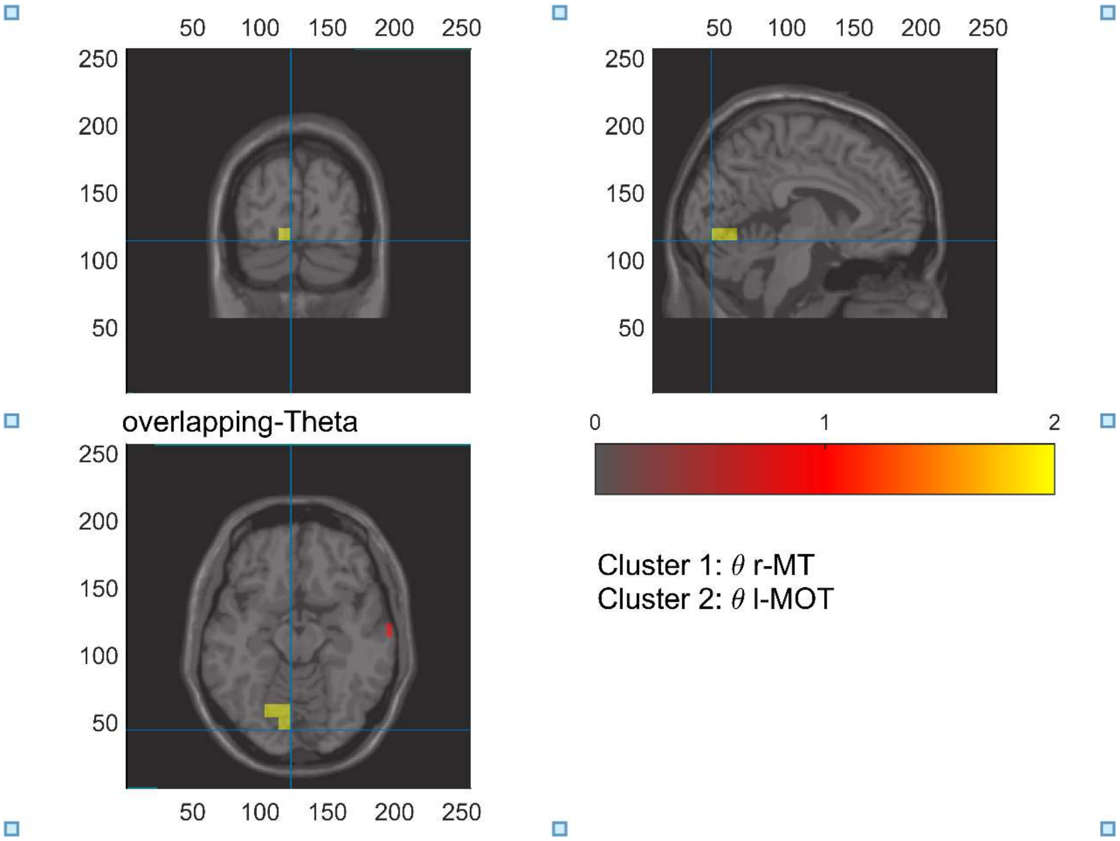

Supplemental Figure S4: Cluster position for the overlapping condition at theta frequency band. Related to STAR Methods as well as Figures 3 and 5. Colors indicate the obtained clusters listed below the color bar.

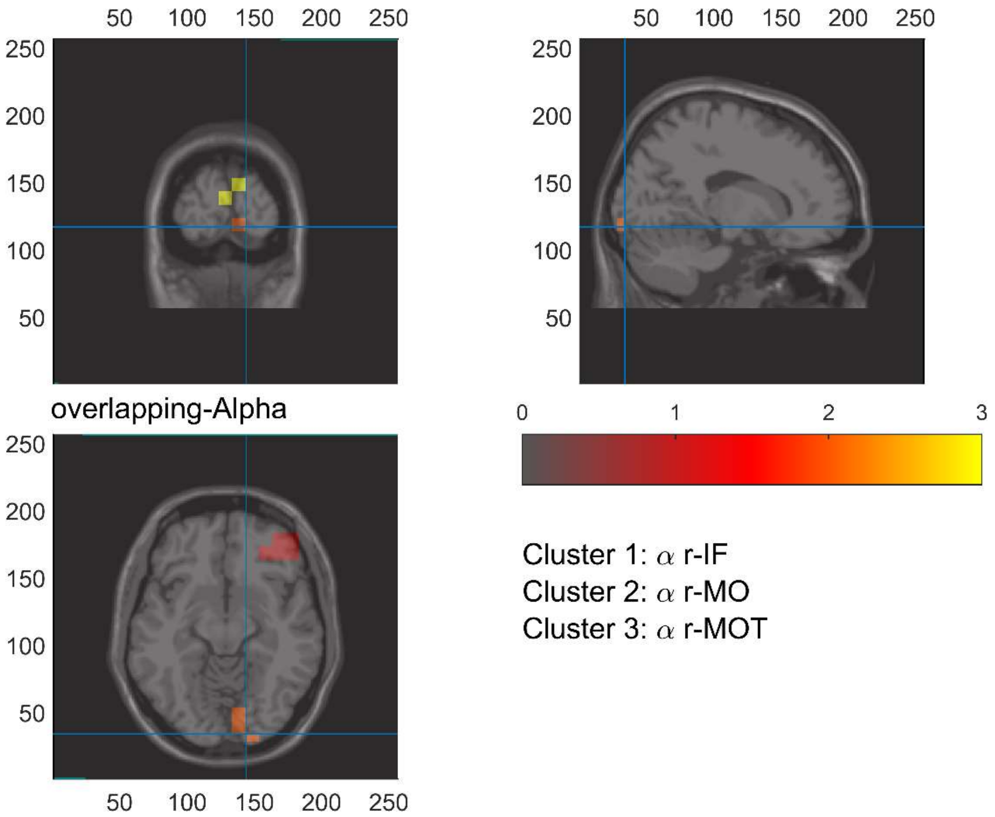

Supplemental Figure S5 Cluster position for the overlapping condition at alpha frequency band. Related to STAR Methods as well as Figures 3 and 5. Colors indicate the obtained clusters listed below the color bar.

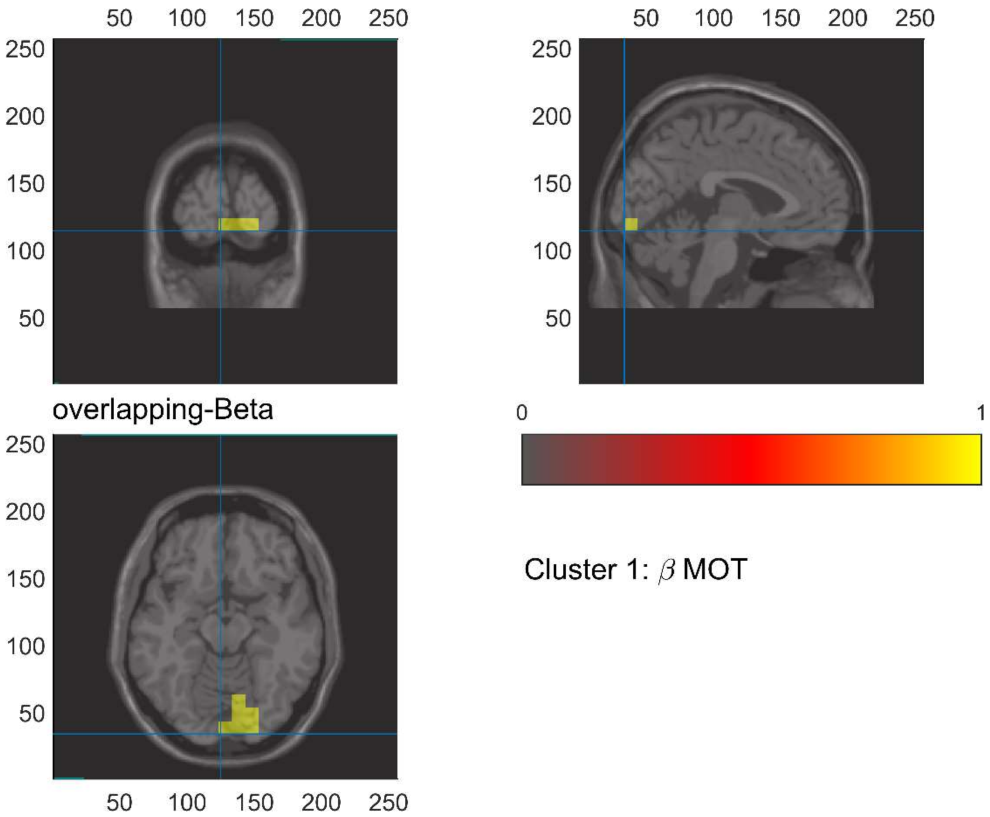

Supplemental Figure S6: Cluster position for the overlapping condition at beta frequency band. Related to STAR Methods as well as Figures 3 and 5. Colors indicate the obtained clusters listed below the color bar.
